# Single-shot all-optical switching of magnetization in Tb/Co multilayer-based electrodes

**DOI:** 10.1038/s41598-020-62104-w

**Published:** 2020-03-23

**Authors:** L. Avilés-Félix, A. Olivier, G. Li, C. S. Davies, L. Álvaro-Gómez, M. Rubio-Roy, S. Auffret, A. Kirilyuk, A. V. Kimel, Th. Rasing, L. D. Buda-Prejbeanu, R. C. Sousa, B. Dieny, I. L. Prejbeanu

**Affiliations:** 1 Spintec, Université Grenoble Alpes, CNRS, CEA, Grenoble INP, IRIG-SPINTEC, 38000 Grenoble, France; 2 Radboud University, Institute for Molecules and Materials, Heyendaalseweg 135, 6525 AJ Nijmegen, The Netherlands; 3 FELIX Laboratory, Radboud University, 7 Toernooiveld, 6525 ED Nijmegen, The Netherlands

**Keywords:** Magnetic devices, Electronic and spintronic devices, Magnetic properties and materials

## Abstract

Ever since the first observation of all-optical switching of magnetization in the ferrimagnetic alloy GdFeCo using femtosecond laser pulses, there has been significant interest in exploiting this process for data-recording applications. In particular, the ultrafast speed of the magnetic reversal can enable the writing speeds associated with magnetic memory devices to be potentially pushed towards THz frequencies. This work reports the development of perpendicular magnetic tunnel junctions incorporating a stack of Tb/Co nanolayers whose magnetization can be all-optically controlled via helicity-independent single-shot switching. Toggling of the magnetization of the Tb/Co electrode was achieved using either 60 femtosecond-long or 5 picosecond-long laser pulses, with incident fluences down to 3.5 mJ/cm^2^, for Co-rich compositions of the stack either in isolation or coupled to a CoFeB-electrode/MgO-barrier tunnel-junction stack. Successful switching of the CoFeB-[Tb/Co] electrodes was obtained even after annealing at 250 ^°^C. After integration of the [Tb/Co]-based electrodes within perpendicular magnetic tunnel junctions yielded a maximum tunneling magnetoresistance signal of 41% and RxA value of 150 Ω*μ*m^2^ with current-in-plane measurements and ratios between 28% and 38% in nanopatterned pillars. These results represent a breakthrough for the development of perpendicular magnetic tunnel junctions controllable using single laser pulses, and offer a technologically-viable path towards the realization of hybrid spintronic-photonic systems featuring THz switching speeds.

## Introduction

Ferrimagnetic systems based on rare earth (RE)-transition metal (TM) alloys and multilayers have been extensively studied in recent decades, largely due to their potential application in the field of magneto-optical recording^1^. The strong perpendicular magnetocrystalline anisotropy inherent to amorphous RE-TM systems have allowed these alloys to play a key role in the historical transition from longitudinal to perpendicular magnetic recording structures^2^, and made them ideal for handling magnetic bit instabilities arising from superparamagnetic effects^3^.

Binary and ternary RE-TM systems such as GdFeCo, GdCo, TbCo or GdFe are still driving forward new developments pertaining to spintronic devices, including spin valves for magnetic read heads^4^, perpendicular magnetic tunnel junctions (p-MTJs)^5,6^ or spin-orbit-torque phenomena^7^. Recent works in this field have also revealed that RE-TM-based films (amorphous or multilayered) represent ideal materials for the observation and study of the phenomena of all-optical switching (AOS)^8–10^. In these systems, it is possible to switch the magnetization using suitable laser pulses without the application of any external magnetic field. Depending on whether the laser-pulses need to be circularly-polarized, AOS of magnetization can be classed as either helicity-dependent (HD-AOS) or helicity-independent (HI-AOS). Furthermore, subject to the material in question, the switching process can be achieved with either a single laser pulse or multiple pulses.

The fundamental mechanisms underpinning all-optical switching still remains a subject of intense debate^11–14^. Nevertheless, the multitude of experimental and theoretical investigations of the AOS processes^15,16^ lead to the reasonable conclusion that not only the laser features (pulse width^11,13^, fluence^17^, number of pulses^9,18^ and polarization^17,19^) but also the properties of the magnetic material^13,16^ determine the switchability of a given system. For example, amorphous GdFeCo alloys exhibit both HD-AOS^8,20^ and HI-AOS^21–23^ in response to single pulses, whereas amorphous TbCo (or FeTb) alloys only display HD-AOS in response to multiple consecutive pulses^9,24^. To date, all RE-free systems (such as CoPt)^25,26^ have only displayed HD-AOS, with the single exception of half-metallic Heusler alloys^14^.

An important milestone in the technological exploitation of AOS^27^ was achieved by Chen *et al*.^5^. In that work, an optically-switchable GdFeCo layer was integrated within a micron-sized p-MTJ pillar showing TMR values of 0.6% upon exposure to a single single ultrashort laser pulse. Unfortunately, Gd-based materials are inherently soft, making it difficult to downscale the size of Gd-based devices below the micrometer length-scales, whereas Tb is capable of supporting domains in continuous films of TbFeCo smaller than 50 nm^28^. Despite the fact that atomistic calculations predict^12^ that single-shot HI-AOS can be achieved in amorphous TbFeCo alloys using femtosecond laser pulses, the practical capability of achieving this in any RE-TM system featuring Tb instead of Gd has, until now, remained undiscovered.

In this article, we report the discovery and development of Tb/Co multilayered systems which allow for single-shot HI-AOS of magnetization. Furthermore, we integrate these optically-switchable Tb/Co multilayered stacks within p-MTJs, yielding TMR values up to 38%. We explore the influence of the annealing temperature on the thermal stability of the multilayers, paying particular attention to the effect on the perpendicular magnetic anisotropy and the M(H) curves. Our experimental results not only demonstrate that it is possible to all-optically switch the magnetization of Tb/Co multilayers - coupled to CoFeB electrodes - using single optical pulses in both as-grown and annealed samples, but also that large TMR values can still be achieved. These results are highly promising for the development of p-MTJs in which the storage layer is all-optically addressed using single-shot laser pulses, thus facilitating writing frequencies that could be advanced towards the THz scale.

## Results

### Perpendicular magnetic anisotropy of [Tb/Co]_*N*_ multilayers

The starting point of our investigation begins with a systematic study of the effect of the nanolayer thickness *t* on the magnetic properties of the Tb/Co multilayered system. We therefore study multilayers of composition [Tb(*t*_Tb_)/Co(*t*_Co_)]_N_ that were fabricated with graded nanolayer thicknesses spanning 6 Å < *t*_Tb_ < 16 Å and 7 Å < *t*_Co_ < 14 Å. The square brackets contain the bilayer structure that is repeated *N* times within each sample. Further details of our sample composition and growth and characterization techniques are supplied in the Methods section.

**Figure 1:**
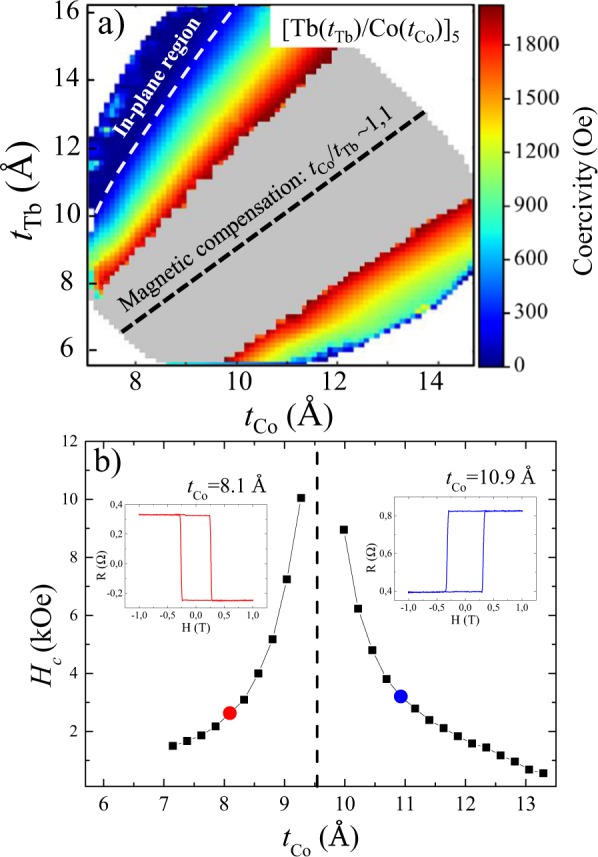
(**a**) Coercive field map of [Tb(*t*_Tb_)/Co(*t*_Co_)]_5_. The coercive field values were obtained from the *M*(*H*) loops measured with *H* applied perpendicular to the plane of the film. The superimposed gray region in the center indicates the region in which *H* was not sufficient to saturate the stack. The black dashed line schematically represents the thickness values of Tb and Co for which the coercive field diverges i.e. where magnetic compensation occurs at room temperature. (**b**) Coercive field of [Tb/Co]_5_ multilayer as a function of Co thickness *t*_Co_: 7–13 Å with the thickness of the Tb layers fixed at *t*_Tb_  =  10 Å. Insets: *R*(*H*) for *t*_Co_  =  8.1 Å and *t*_Co_  = 10.9 Å as indicated.

Coercive field maps obtained by measuring hysteresis loops on a spatially-resolved basis across the crossed-wedge samples enabled us to identify nanolayer thickness combinations that supported the formation of uniaxial magnetic anisotropy with an easy axis perpendicular to the film’s plane. The maximum magnetic field that can be applied in our Kerr set-up is 2.4 kOe. Fig. 1a shows a typical mapping of the coercive field across the sample featuring *N*  =  5 repetitions of [Tb/Co], whereby red and blue areas indicate regions with a strong and weak coercive field respectively. The grey region in the center of the map corresponds to the region in which the external magnetic field *H* was incapable of reversing the magnetization of the [Tb/Co]_*N*_ multilayer stack. As can be seen in the figure, the coercive field diverges as the composition approaches the thickness ratio *t*_Co_/*t*_Tb_ ~  1.1. The black dashed line superimposed over Fig. 1a corresponds to this ratio, and lies reasonably equidistant between the two measurable regions. These considerations indicate that the magnetic moment compensation of the Co and Tb sublattices occurs near the central part of the gray region shown in Fig. 1a.

Complimentary to the coercive field mapping, resistance loops *R*(*H*) were acquired using an Extraordinary Hall Effect (EHE) setup. An additional Co-single-wedge sample with a fixed Tb-layer thickness *t*_Tb_ = 10 Å was used to obtain a more detailed dependence of the coercive field as a function of *t*_Co_. Insets of Fig. 1b are typical measurements obtained for *t*_Tb_  =  10 Å with two different Co-layer thicknesses *t*_Co_ as indicated. The clear inversion of the *R*(*H*) loops (with respect to *H*) for the different *t*_Co_ thicknesses shows the existence of an intermediate thickness that allows for the total compensation of the magnetization. We therefore repeated the measurements with varying *t*_Co_, and the results are summarized in the main panel of Fig. 1b. We also observed a slight difference in the Co thickness required to achieve magnetic compensation in the coercivity mapping and in the single-wedge multilayer. The origin of the 1.5 Å-shift in the Co-thickness (*t*_Co_ = 9.5 Å in Fig. 1b and *t*_Co_ = 11 Å in the coercivity mapping) to achieve compensation with *t*_Tb_ = 10 Å, is attributed to a growth-induced anisotropy that modifies the coercive field of the multilayer at room temperature.

### Thermal stability of the Tb/Co multilayers

While Tb/Co multilayers represent ideal candidates for integration within p-MTJs, several investigations have highlighted the fact that the magnetic properties of Tb/Co-based systems, such as the anisotropy coefficient or the coercive field, can be substantially affected by post-deposition annealing^29,30^, even at relatively low temperatures ( ≈ 200 ^°^C)^31^. This has significant implications for the technological usefulness of Tb/Co multilayered stacks, and must therefore be investigated.

**Figure 2:**
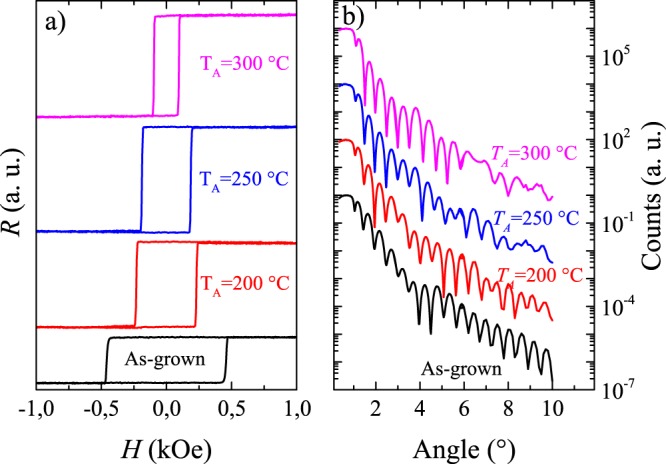
(**a**) *R*(*H*) obtained from EHE experiments and (**b**) x-ray reflectivity measurements of [Tb(10 Å)/Co(12 Å)]_5_ samples as-deposited and annealed at temperatures *T*_*A*_  =  200, 250 and 300 ^°^C as indicated.

The influence of the annealing temperature (*T*_*A*_) on the magnetic properties of the stack [Tb(10 Å)/Co(12 Å)]_5_ with uniform thicknesses is shown in Fig. 2. Figure 2a clearly shows that as the annealing temperature is increased, the coercive field decreases. This change of coercitivity strongly depends on the ratio *t*_Co_/*t*_Tb_, and is associated with different structural transformations that occur at the interface during the annealing^29^. XRR measurements (Fig. 2b), also performed for the stack [Tb(10 Å)/Co(12 Å)]_5_ with varying *T*_*A*_, also evidences a substantial loss in the number of oscillations for angles between 6^°^ and 10^°^. Furthermore, there is a substantial change in the amplitude of oscillation of the XRR pattern, which is similarly attributed to an enhancement of the interfacial roughness between the nanolayers originating from interdiffusion or even structural relaxation modifying the strain within the sample. This degradation of the interfaces, which clearly occurs for post-deposition annealing temperatures above 200 ^°^C, can be correlated with the weakening of the perpendicular anisotropy.

### Single-shot all-optical switching of CoFeB-[Tb/Co]_*N*_ electrodes

**Figure 3:**
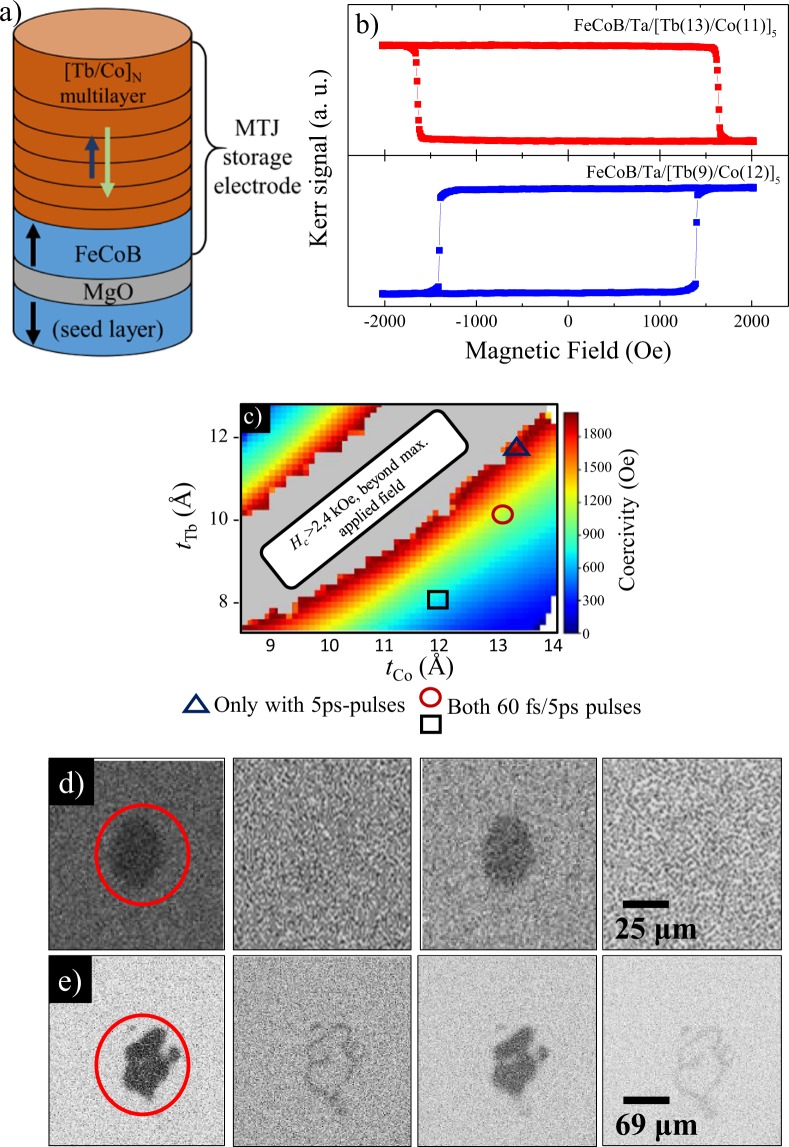
(**a**) Structure of half-MTJ stack used to optimize the magnetic coupling between the [Tb(10 Å)/Co(12 Å)]_5_ multilayer and the CoFeB electrode. (**b**) Hysteresis loop of the CoFeB/Ta/[Tb(*t*_Tb_)/Co(*t*_Co_)]_5_ stack measured on opposite sides of the compensation region. (**c**) Coercivity map of the half-MTJ along the Tb and Co thickness wedges. The regions enclosed by the symbols indicate the three regions of the CoFeB/Ta/[Tb(*t*_Tb_)/Co(*t*_Co_)]_5_ structure in which all-optical switching was achieved using different pulse durations: ▵: [Tb(11 Å)/Co(13 Å)]_5_, ○: [Tb(10 Å)/Co(13 Å)]_5_ and □: [Tb(8 Å)/Co(12 Å)]_5_ as indicated. (**d**–**e**) Background-corrected magneto-optical images demonstrating successful single-shot all-optical switching of magnetization in the CoFeB/Ta/[Tb(10 Å)/Co(13 Å)]_5_ stack using (**d**) ps- and **e**) fs-long laser pulses in with an incident fluence of 4.7 mJ/cm^2^ and 3.5 mJ/cm^2^ respectively.

To integrate the [Tb/Co]_*N*_ system as part of the storage layer of a perpendicular magnetic tunnel junction, we coupled the crossed-wedge multilayer with a CoFeB electrode through an ultrathin Ta layer, thus fabricating the MTJ electrode seed-layer//MgO/CoFeB(13 Å)/Ta(2 Å)/[Tb(*t*_Tb_)/Co(*t*_Co_)]_5_. A schematic of the MTJ storage electrode is shown in Fig. 3a. This sample (both as-deposited and annealed at 250 ^°^C) was characterized using Kerr magnetometry. In both cases, the compensation point remains around the same thickness ratio *t*_Co_/*t*_Tb_ ~ 1.1 as for the case of the isolated [Tb(*t*_Tb_)/Co(*t*_Co_]_5_ multilayer.

The *M*(*H*) loops shown in Fig. 3b, extracted from the coercitivity map of the crossed-wedge structures annealed at 250 ^°^C, present only one step in magnetization, indicating a relatively strong coupling between the 13 Å-thick CoFeB layer and the [Tb/Co]_5_ stack that persists after annealing. The type of magnetic coupling (ferromagnetic or antiferromagnetic) between the [Tb/Co]_*N*_ synthetic ferrimagnet and the ferromagnetic CoFeB electrode was taken into account during the design of the storage layer. Depending on whether the initial layer of the [Tb/Co]_*N*_ stack is Tb or Co, the interlayer exchange coupling between the CoFeB and the stack can be antiferromagnetic or ferromagnetic respectively. Our MTJ electrode deliberately couples the CoFeB directly to the Tb sublattice in order to maintain an antiferromagnetic coupling in our stack. Further discussion related to the influence of the initial layer of the [Tb/Co]_*N*_ on the magnetic properties of the electrodes can be found in the supplementary data file.

It is important to note that the magnetic coupling between the ferromagnetic electrode and the synthetic ferrimagnetic multilayered stack was achieved for both Tb-rich and Co-rich regions in as-deposited samples and annealed at *T*_*A*_  =  250^ °^C. Part of the coercive field map shown in Fig. 3c reveals that neither the presence of the additional CoFeB layer nor the applied annealing significantly shift the compensation region of the half-MTJ.

Single-shot HI-AOS of the magnetization in the CoFeB-[Tb(*t*_Tb_)/Co(*t*_Co_)] electrode was explored in three different regions of the crossed-wedge sample after annealing, using either fs-long or ps-long laser pulses. The different regions of the MTJ electrode (annealed at *T*_*A*_  =  250 ^°^C) that exhibited HI-AOS are indicated in the coercitivity mapping of Fig. 3c: [Tb(8 Å)/Co(12 Å)]_5_, [Tb(10 Å)/Co(13 Å)]_5_ and [Tb(12 Å)/Co(13 Å)]_5_. The margin error of *t*_Tb_ and *t*_Co_ is 1 Å. Interestingly, while both 60 fs-long and 5 ps-long pulses were capable of single-handedly toggling the magnetization in CoFeB/[Tb(8 Å)/Co(12 Å)]_5_ and CoFeB/[Tb(10 Å)/Co(13 Å)]_5_, the region with larger thicknesses CoFeB/[Tb(11 Å)/Co(13 Å)]_5_ could only be optically-addressed using 5 ps-long pulses. The 60 fs-long pulses only served to demagnetize this region.

The HI-AOS of magnetization was achieved for CoFeB/[Tb(10 Å)/Co(13 Å)]_5_ with an incident fluence down to 4.7 mJ/cm^2^ and 3.5 mJ/cm^2^ using ps-long and fs-long laser pulses respectively and only for Co-rich compositions, in which the compensation temperature is below room temperature, showed HI-AOS. At other regions of the sample, the optical pulses only demagnetized the sample.

Figure 3d presents background-corrected Kerr microscopy images obtained after exposing the CoFeB/Ta/[Tb(10 Å)/Co(13 Å)]_5_ electrodes to consecutive 5 ps-long laser pulses with an incident fluence^32^ of 4.7 mJ/cm^2^. The total energy of each laser pulse was 138 nJ (estimated by measuring the power of laser pulses with 1 kHz repetition rate). On the other hand, the images in Fig. 3e correspond to the reversal of the magnetization using fs-long laser pulses with a fluence of 3.5 mJ/cm^2^. The HI-AOS process in the MTJ electrode occurs in both the CoFeB and in the [Tb/Co]_*N*_ system due to the strong magnetic coupling between the CoFeB and the Tb/Co multilayer as evidenced by the *M*(*H*) loops. Further discussion about the pulse duration and fluence dependence of the switching in the CoFeB/Ta/[Tb/Co]_5_ can be found in the supplementary file. The magneto-optical response was also tested in samples with *N* = 15 repetitions of the [Tb/Co]_*N*_ multilayer, but the required incident fluence needed to reverse the magnetization was 4 times higher ( ~19 mJ/cm^2^) compared to that required with *N*  =  5.

### TMR measurements of nanopatterned MTJ with the optically-switchable electrodes

**Figure 4:**
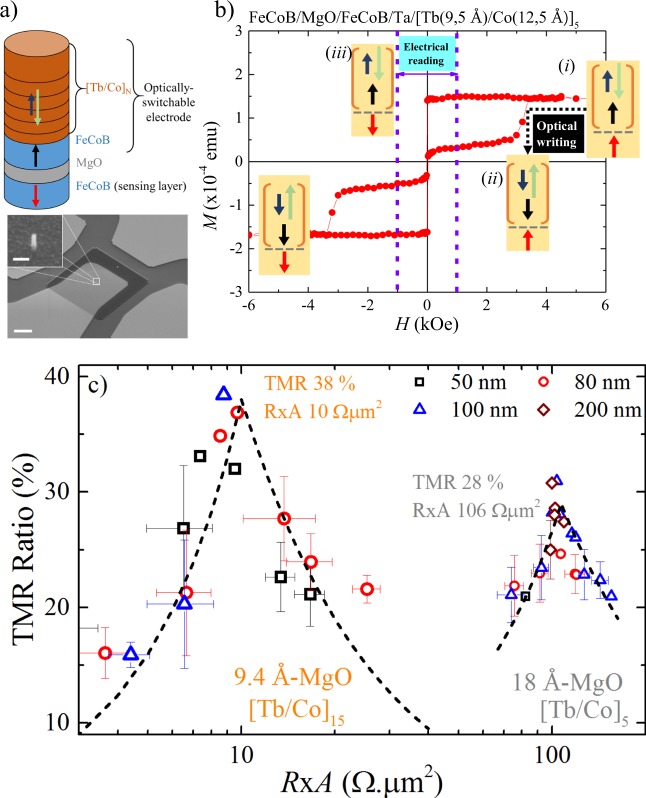
(**a**) Top: Schematic of the MTJ studied. The MTJ consists of an optically-switchable storage layer CoFeB (13Å)/Ta/[Tb/Co]_*N*_ and a CoFeB free layer, separated by a MgO tunnel barrier. Bottom: SEM Image of the 50 nm patterned MTJ in an intermediate step of the nanofabrication process. (scale bar: 4 *μ*m). Inset: Zoom of the capping layer of the 50 nm-junction before the deposition of the upper electrical contact (scale bar: 100 nm) (**b**) Out-of-plane *M*(*H*) curve measured for the MTJ with the stack [Tb/Co]_5_. Insets: illustrations of the 4 magnetic states of the MTJ indicating the stages intended for optical writing (state (*i*) to state (*ii*)) and electrical readout (state (*i*) to state (*iii*)). (**c**) TMR vs RxA of nano-patterned junctions for stacks with different repetition numbers *N* of the [Tb/Co]_*N*_ stacks as indicated, with 9.4 Å and 18 Å MgO barriers. The dispersion in the TMR and RxA as calculated from shunt and series resistance models (dashed lines) are also shown. The wafer-level TMR potential for optimal processing is 38% and 28% respectively.

Conventional p-MTJ structures typically have a CoFeB free layer and a synthetic antiferromagnet (SAF) reference layer based on (Co/Pt) multilayers coupled to a CoFeB electrode. The SAF reference layer has typical coercive fields on the order of 1-3 kOe, similar to those expected from the optically-switchable CoFeB-[Tb/Co]_*N*_ electrode. To clearly distinguish the reversal from these two electrodes, we fabricated p-MTJ devices with an AOS-compatible electrode coupled to a free CoFeB counter-electrode, with the latter acting as a sensing layer for electrical readout. A schematic of the considered all-optically-switchable MTJ structure is shown in the top part of Fig. 4a.

The complete structure of the magnetic tunnel junction containing the optically switchable electrode is: Ta(30Å)/FeCoB(11Å)/MgO/FeCoB(13Å)/Ta(2Å)/[Tb/Co]_*N*_. Fig. 4b shows the out-of-plane hysteresis loop measured for the full MTJ annealed at *T*_*A*_  =  250 ^°^C. The p-MTJ structure incorporates both the optically-addressable electrode CoFeB/Ta/[Tb(9.5 Å)/Co(12.5 Å)]_5_ and a 11 Å-thick CoFeB layer serving as the sensing layer. The insets of Fig. 4b present a schematic of the 4 possible magnetic configurations of the full all-optically-switchable MTJ. During the process of all-optical writing, the magnetization of the AOS electrode will switch from state (*i*) to state (*ii*) (black dotted arrow), corresponding to a change from parallel to antiparallel magnetic states in the MTJ. To evaluate the TMR values, current-in-plane tunneling (CIPT) measurements were performed, in which we electrically reversed the magnetization of the single CoFeB sensing layer in our p-MTJ i.e. passing from state (*i*) to state (*iii*). The TMR and RxA values measured in fullsheet samples were 30% and 19 Ω*μ*m^2^ respectively for [Tb(8.7 Å)/Co(13 Å)]_15_, and 41% and 150 Ω*μ*m^2^ for [Tb(9.5 Å)/Co(12.5 Å)]_5_ using CIPT measurements. On the other hand, electrical evaluation of the nanopatterned AOS-MTJ pillars indicate the viability of obtaining similar TMR values even after the nanofabrication process. Fig. 4c shows the distribution of TMR vs R × A values in nanopatterned CoFeB/MgO/CoFeB(13 Å)/Ta/[Tb/Co]_*N*_ pillars of different junction diameters. The MTJs were fabricated with 9.4 Å-thick and 18 Å-thick MgO barriers for *N*  = 15 and 5 repetitions of the [Tb/Co] bilayers respectively. The black dashed lines correspond to the distributions of TMR assuming shunt and series resistance defects in patterned pillars. The maximum potential TMR for optimal processing is 38 % and 28% for *N*  =  15 and 5 repetitions of the [Tb/Co] respectively. These values of TMR obtained in our Tb/Co-based optically-addressable MTJs clearly demonstrate that this multilayer system is an excellent candidate for integration within hybrid photonic-spintronic devices. Additional experimental data corresponding to the TMR distribution of the nanopatterned junctions of this sample have been included in the Supplementary data file.

## Discussion

We have fabricated nanopatterned magnetic tunnel junctions exhibiting TMR values of about 38% and 28% for *N*  =  15 and 5 repetitions of the [Tb/Co] respectively, using optically-switchable CoFeB/Ta/[Tb/Co]_*N*_ multilayers as the storage electrode. Through adjusting the combination of thicknesses of the Tb and Co nanolayers, we are able to achieve full control over both the coercive field and the out-of-plane anisotropy of the synthetic ferrimagnet. Annealing of the multilayered stack at various temperatures allowed us to evaluate the thermal stability of the stack’s magnetic properties. In order to fabricate an optically-switchable storage layer, we have coupled a CoFeB single layer and the multilayered [Tb/Co] stack. Single-shot all-optical switching of the storage layer’s magnetization was achieved using both 60 fs-long and 5 ps-long laser pulses, with incident fluences down to 3.5 mJ/cm^2^. Importantly, we reveal that the all-optical switching only occurs for [Tb/Co] multilayers for different thicknesses in the Co-rich composition in the FeCoB-[Tb/Co]_*N*_ electrodes. The thermal stability of the AOS-MTJ is maintained even after annealing at 250 ^°^C, presenting a TMR signal of 41% and RxA value of 150 Ω*μ*m^2^ in CIPT measurements and up to 38% in nanopatterned pillars.

On the one hand, our research demonstrates the viability of integrating optically-switchable materials in to MTJs and still obtain high TMR values. On the other hand, the observation of single-shot HI-AOS in a [Tb/Co] multilayered system, and its integration within a MTJ compatible with post-deposition annealing, represents a significant breakthrough both in the fields of ultrafast magnetism and device physics. As well as stimulating investigations into the physics underpinning the HI-AOS process, these results will undoubtedly contribute to the development of magnetic random access memories with new functionalities and potentially ultrafast speeds.

## Methods

To systematically study the effect of the nanolayer thickness on the magnetic properties of the Tb/Co multilayered system, 4” multilayers of composition [Tb(*t*_Tb_)/Co(*t*_Co_)]_N_ have been fabricated with graded thicknesses of the Tb and Co layers spanning 6 Å < *t*_Tb_ < 16 Å and 7 Å < *t*_Co_ < 14 Å. The square brackets contain the bilayer structure that is repeated *N* times within each sample. A 30 Å-thick Ta layer and a Cu(20 Å)/Pt(30 Å) bilayer were used as a buffer and capping layers respectively. Samples were grown on thermally-oxidized single crystal Si(100) wafers by DC magnetron sputtering using an argon pressure of 2 mbar and a base pressure of 10^−8^ mbar.

Hysteresis loops were measured using an Extraordinary Hall Effect (EHE) setup, and coercive field (*H*_*c*_) spatially-resolved mappings were acquired using a Kerr magnetometer in polar configuration. The maximum magnetic field that can be applied in our Kerr set-up is 2.4 kOe. Verification of the multilayer thickness and the influence of the annealing on the structural properties of the multilayers was performed by measuring the periodicity and intensity of the Kiessig fringes extracted from low-angle X-Ray reflectivity (XRR) measurements.

To visualize magnetic domains in the multilayers, we used a magneto-optical imaging microscope in the polar Kerr configuration. All-optical switching was tested using optical pulses (of central wavelength 800 nm and duration tunable between 60 fs and 5 ps) sourced from a pulsed-amplified-laser system capable of generating laser pulses on a single-shot basis.

## Supplementary information

Supplementary Information.
