# Supplementary material for: Single-shot all-optical switching of magnetization in Tb/Co multilayer-based electrodes
Source: Sci Rep. 2020 Mar 23;10:5211. doi: 10.1038/s41598-020-62104-w (PMC7089968; doi:10.1038/s41598-020-62104-w)
Supplement: Supplementary file 1 — Supplementary Information. [file 41598_2020_62104_MOESM1_ESM.pdf]

Single-shot all-optical switching of magnetization in Tb/Co multilayer-based magnetic tunnel junction electrodes

L. Avilés-Félix, A. Olivier, G. Li, C. S. Davies, L. Alvaro-Gómez, M. Rubio-Roy, S. Auffret, A. Kirilyuk, A. V. Kimel, Th. Rasing, L. D. Buda-Prejbeanu, R. C. Sousa, B. Dieny and I. L. Prejbeanu

[1]Spintec, Université Grenoble Alpes, CNRS, CEA, Grenoble INP, IRIG-SPINTEC, 38054 Grenoble, France
[2]Radboud University, Institute for Molecules and Materials, Heyendaalseweg 135, 6525 AJ Nijmegen, The Netherlands
[3]FELIX Laboratory, Radboud University, 7 Toernooiveld, 6525 ED Nijmegen, The Netherlands
*Corresponding author:luis.avilesfelix@cea.fr

Supplementary data

S1. Mean atomic concentration of the $[Tb/Co]_N$ multilayered system to achieve magnetic compensation.

It is possible to determine the mean atomic concentrations of the Tb and Co layers [Ref S1] required to compensate the magnetic moments of the sublattices at room temperature, by considering the atomic mass and the densities of Tb and Co.

$$Atomic\ percentage\ of\ Tb = \frac{t_{Tb}}{t_{Tb} + 2.92\ t_{Co}}$$

The values t_{Tb}=10 Å and t_{Co}= 9.5 Å implies the relative atomic proportion of Co and Tb is 73.5 % and 26.5% respectively. Our estimation of the atomic concentration of the compensation thicknesses agrees with previous values reported in other works [Ref S2, Ref S3] who obtained 72.2 % of Co in a multilayered system and 78 % of Co in an amorphous alloy of Tb_xCo_{1-x}. It is important to mention that in our system, not only the perpendicular magnetic anisotropy (PMA) but also the optical-switchability strongly depend on the relative concentrations of Tb and Co. Although the ratio t_{Co}/t_{Tb} ≈ 1.1 yields a strong PMA, reduction of the ratio t_{Co}/t_{Tb} caused the easy axis of the uniaxial anisotropy to rotate in-plane, as indicated in Fig. 1a of the manuscript.

S2. Additional data to discuss the influence of the initial layer on the magnetic properties of the optically switchable electrodes.

Our magnetic tunnel junction electrode consists of a [Tb/Co] multilayer coupled to a FeCoB layer separated by an ultrathin layer of Ta. As know, in a ferromagnetic multilayer as $[Tb/Co]_5$ the coupling between the Tb and Co atoms at the interface is antiferromagnetic. It is also expected an antiferromagnetic coupling between transition metal atoms of the FeCoB layer and the Tb atoms. To explore this, we characterize three different structures: the isolated $[Tb/Co]_5$ multilayer, FeCoB/Ta/$[Tb/Co]_5$ and FeCoB/Ta/$[Co/Tb]_5$ structures. Fig. S1a shows the coercive field mapping of an isolated $[Tb/Co]_5$ multilayer, in which the compensation region

(blue fringe) is located at the center of the wafer. Vertical and horizontal lines located at t_{Tb}=12 Å and t_{Co}=10Å are guides to the eyes. The hysteresis loops obtained during the mapping showed high remanence and a square shape evidencing the anisotropy easy axis perpendicular to the plane. The growth of an additional FeCoB layer to form the optical switching electrode evidenced that the coupling between the FeCoB and the initial layer of the ferrimagnetic multilayer has a significant impact on the compensation region. Fig S1b and S1c presents the coercive field mapping of the FeCoB/Ta/[**Tb**/Co]$_5$ and FeCoB/Ta/[**Co**/Tb]$_5$ structures respectively. The seed layer consist of a Ta(30Å)/FeCoB(4Å)/MgO(12Å) structure. Coercivity mappings were obtained from the as-deposited samples. As can be seen from the mapping of the sample with Tb as adjacent layer to the FeCoB (Fig. S1b), the presence of the FeCoB has no a significant impact in the thicknesses required to achieve compensation; however, from the mapping of the sample with Co as adjacent layer to the FeCoB (Fig. S1c), we evidenced that the stability of the perpendicular anisotropy of the Tb-rich region is strongly affected and the thicknesses required to achieve compensation can't be determined. Hysteresis loop extracted from figures S1b and S1c for t_{Tb}=12.4 Å and t_{Co}=8.1 Å also showed that the Kerr signal was inverted with respect to the external magnetic field (Fig. S1e), indicating that the interlayer coupling was also affected by the FeCoB-Co exchange coupling.

Finally, Fig. S1d shows the hysteresis loops of the electrodes with t_{Tb}=10.5 Å and t_{Co}=13.2 Å for the FeCoB/Ta/[Tb/Co]$_5$ electrode. Although, the coercive field of the samples with Tb as initial layer increases a 60 % with respect to its counterpart, the perpendicular anisotropy is kept for the thicker Co regions, and more important, the PMA is also kept after annealing.

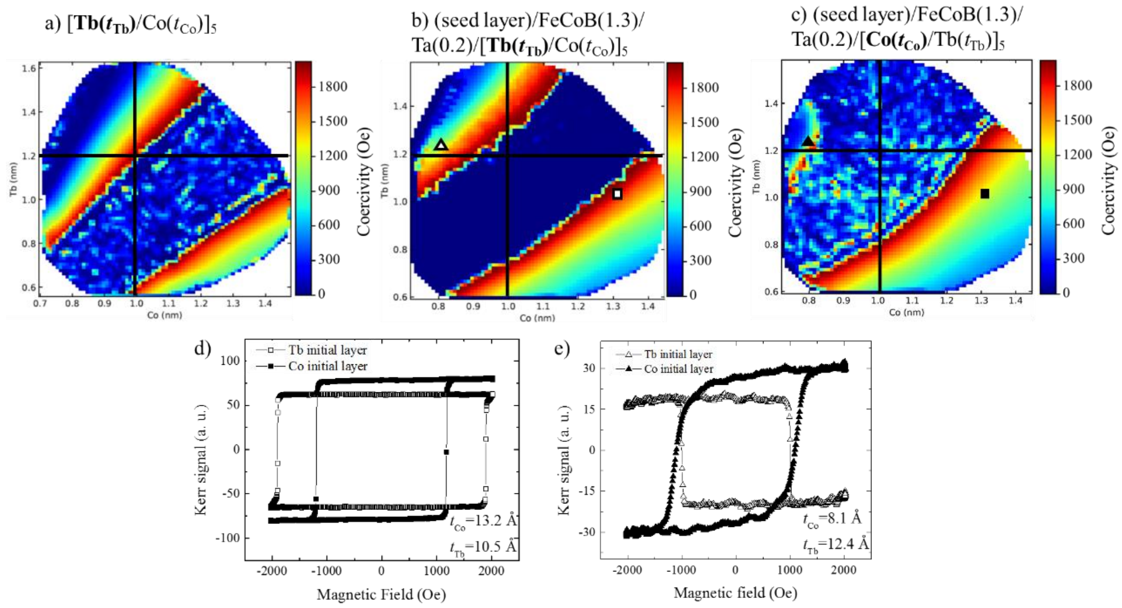

Fig S1. Coercive field mapping of a)[Tb/Co]$_5$ multilayers, b)FeCoB/Ta/[Tb/Co]$_5$ and c)FeCoB/Ta/[Co/Tb]$_5$ structures to explore the influence of the initial layer of the [Tb/Co] multilayers within the tunnel junction electrodes. Vertical and horizontal lines located at t_{Tb}=12 Å and t_{Co}=10Å are guides to the eyes. The seed layer consist of a Ta(30Å)/FeCoB(4Å)/MgO(12Å) structure, in which the 4Å-FeCoB is a dead magnetic layer. c) and d) Kerr hysteresis loop of the electrodes with Tb (□,△) and Co (■,▲) as adjacent layer to the FeCoB with d) t_{Tb}=10.5 Å and t_{Co}=13.2 Å and e) t_{Tb}=12.4 Å and t_{Co}=8.1 Å. Hysteresis loops were extracted from the coercive field mapping showed in b) and c).

S P I N T E C
(Univ. Grenoble Alpes, CEA, CNRS – UMR8191)
Building 10.05 – 17, rue des Martyrs – CEA Grenoble – 38054 GRENOBLE – FRANCE
Phone : +(33) 4.38.78.49.77 – Fax : +(33) 4.38.78.21.27
direction.spintec@cea.fr | http://www.spintec.fr

S3. Additional data to discuss the pulse duration and fluence dependence of the all-optical helicity independent switching in CoFeB-$[Tb/Co]_5$ electrodes.

The laser pulse duration and fluence dependence for CoFeB/$[Tb(10\ Å)/Co(13\ Å)]_5$ is shown in Fig. S2 (next page) using single 70 fs and 7 ps laser pulses with fluences F = 3.1, 3.5 and 4.0 mJ/cm^2. As can be seen from Fig. S2 images obtained for a laser pulse duration D = 7 ps, a clear reverse of the magnetization is observed for F = 3.1 mJ/cm^2. Fluences above 3.5 mJ/cm^2 induce thermal demagnetization regions at the center of the induced magnetic domain and fully reversal of the magnetization near the edges of the spot. The demagnetized regions are enclosed by a purple dashed circle in Fig.S2. By reducing the pulse duration to 70 fs, the fluence required to induce thermal demagnetization on the sample increases. In contrast with D = 7ps, 70 fs allow us to reverse the magnetization for 3.5 and 4.0 mJ/cm^2. The optimal fluence window to observe HI-AOS in FeCoB-$[Tb/Co]_N$ shifts towards lower energies as we increase the pulse duration of the single pulse.

S4. TMR distribution of FeCoB(11Å)/MgO(23Å)/FeCoB(13Å)/Ta(2Å)/$[Tb(9.5Å)/Co(12.5Å)]_5$ nanopatterned junctions.

The TMR distribution of hundreds of MTJ with different diameters is shown in Fig. S3a. The full structure of the junctions consist of: Ta(30Å)/FeCoB(11 Å)/MgO(23 Å)/FeCoB(13 Å)/Ta(2 Å)/$[Tb(9.5\ Å)/Co(12.5\ Å)]_5$. The maximum TMR ratio (36 %) was observed for a 200 nm-diameter junction with a minimum resistance of 6 kΩ. Fig. S3a shows the corresponding resistance loop extracted from the 200 nm-diameter (yellow) distribution of Fig. S3a.

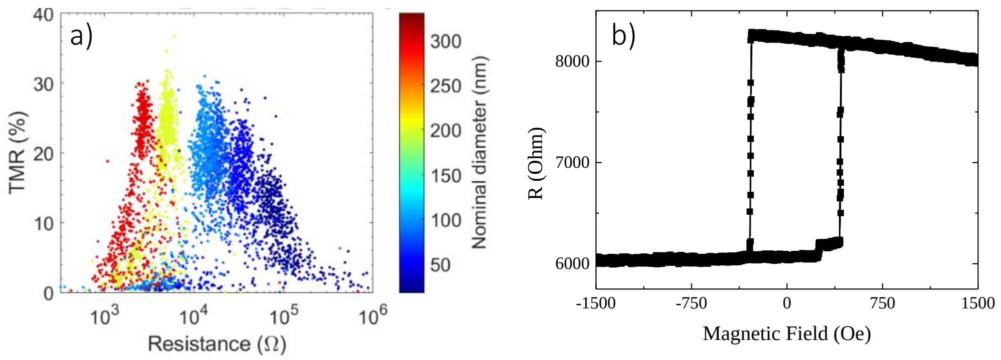

Fig S3. a) TMR distribution of the Ta(30Å)/FeCoB(4Å)/MgO(23Å)/FeCoB(13 Å)/Ta(2 Å)/$[Tb(9.5\ Å)/Co(12.5\ Å)]_5$ nanopatterned junction for different diameters. b) Resistance loop of a 200 nm-diameter with a TMR ratio of 36 %.

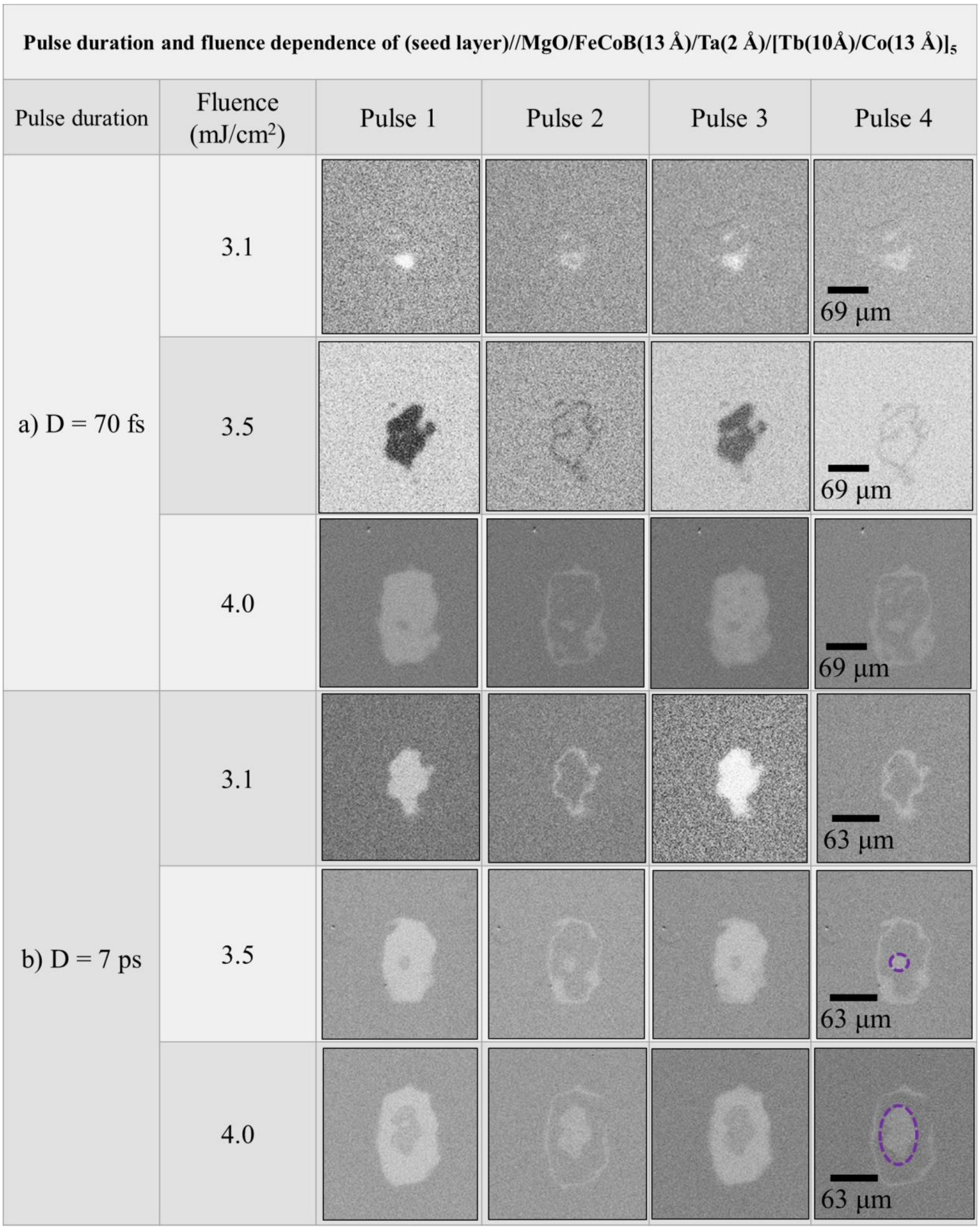

Fig S2. Magneto-optical images demonstrating the pulse duration and fluence dependence of the magnetization reversal in (seed layer)//MgO/FeCoB(13 Å)/Ta(2 Å)/[Tb(10 Å)/Co(13 Å)]$_5$. Images were taken after the incidence of single 70 fs and 7 ps laser pulses with fluences F = 3.1, 3.5 and 4.0 mJ/cm^2. Purple dashed circles indicate the regions that showed clear thermal demagnetization after the pulse.

S5. Hysteresis loop of CoFeB/MgO/CoFeB/Ta/[Tb(10 Å)/Co(13 Å)]$_{15}$.

During the characterization of the optically-switchable electrodes we also explored the magnetic properties and magneto-optical response of the [Tb/Co] system as a function of the number of repetitions in the multilayer. Crossed-wedge samples with 15 repetitions of [Tb/Co]

UNIVERSITÉ Grenoble Alpes
S P I N T E C
(Univ. Grenoble Alpes, CEA, CNRS – UMR8191)
Building 10.05 – 17, rue des Martyrs – CEA Grenoble – 38054 GRENOBLE – FRANCE
Phone : +(33) 4.38.78.49.77 – Fax : +(33) 4.38.78.21.27
direction.spintec@cea.fr | http://www.spintec.fr

not only showed strong PMA and response after the incidence of single laser pulses, but also high values of TMR after processing. Fig. S4 shows the M(H) of a sample with uniform thickness and with 15 repetitions of the Tb/Co multilayer after its integration within a MTJ structure.

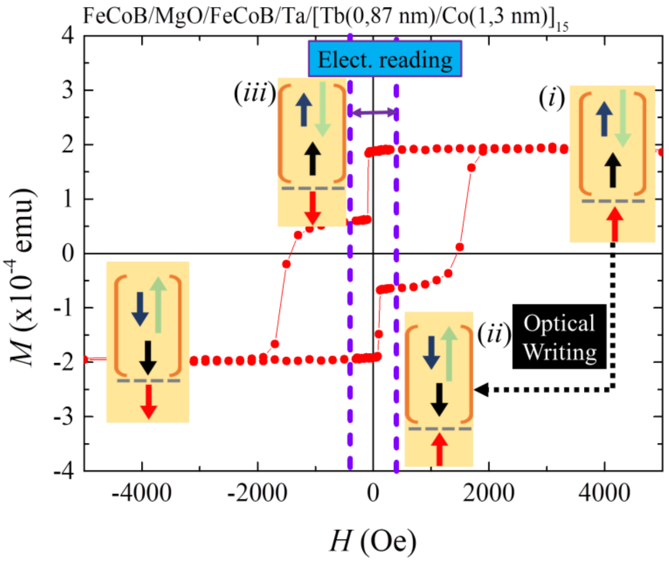

Fig S4. Out-of-plane *M*(*H*) curve measured for the MTJ with the stack $[Tb/Co]_{15}$. Inset: Illustration of the 4 magnetic states indicating the stages intended for optical writing and electrical readout.

References

Ref S1. Richomme, F., Teillet, J., Fnidiki, A., Auric, P. & Houdy, P. Experimental study of the structural and magnetic properties of Fe/Tb multilayers. *Phys. Rev. B* **54**, 416–426, DOI: 10.1103/PhysRevB.54.416 (1996).

Ref S2. Frackowiak, Ł., Kuswik, P., Urbaniak, M., Chaves-O'Flynn, G. D. & Stobiecki, F. Wide-range tuning of interfacial exchange coupling between ferromagnetic Au/Co and ferrimagnetic Tb/Fe(Co) multilayers. *Sci. Reports* **8**, 16911, DOI:1038/s41598-018-35042-x (2018).

Ref S3. Gottwald, M. et al. Magnetoresistive effects in perpendicularly magnetized tb-co alloy based thin films and spin valves. *J. Appl. Phys*. **111**, 083904, DOI: 10.1063/1.3703666 (2012).
